# Generating Potential RET-Specific Inhibitors Using a Novel LSTM Encoder–Decoder Model

**DOI:** 10.3390/ijms25042357

**Published:** 2024-02-17

**Authors:** Lu Liu, Xi Zhao, Xuri Huang

**Affiliations:** Institute of Theoretical Chemistry, College of Chemistry, Jilin University, Changchun 130061, China; l_liu18@mails.jlu.edu.cn

**Keywords:** RET, deep learning, encoder–decoder network, long short-term memory (LSTM), virtual screening, molecular dynamics simulation

## Abstract

The receptor tyrosine kinase RET (rearranged during transfection) plays a vital role in various cell signaling pathways and is a critical factor in the development of the nervous system. Abnormal activation of the RET kinase can lead to several cancers, including thyroid cancer and non-small-cell lung cancer. However, most RET kinase inhibitors are multi-kinase inhibitors. Therefore, the development of an effective RET-specific inhibitor continues to present a significant challenge. To address this issue, we built a molecular generation model based on fragment-based drug design (FBDD) and a long short-term memory (LSTM) encoder–decoder structure to generate receptor-specific molecules with novel scaffolds. Remarkably, our model was trained with a molecular assembly accuracy of 98.4%. Leveraging the pre-trained model, we rapidly generated a RET-specific-candidate active-molecule library by transfer learning. Virtual screening based on our molecular generation model was performed, combined with molecular dynamics simulation and binding energy calculation, to discover specific RET inhibitors, and five novel molecules were selected. Further analyses indicated that two of these molecules have good binding affinities and synthesizability, exhibiting high selectivity. Overall, this investigation demonstrates the capacity of our model to generate novel receptor-specific molecules and provides a rapid method to discover potential drugs.

## 1. Introduction

**Figure 1:**
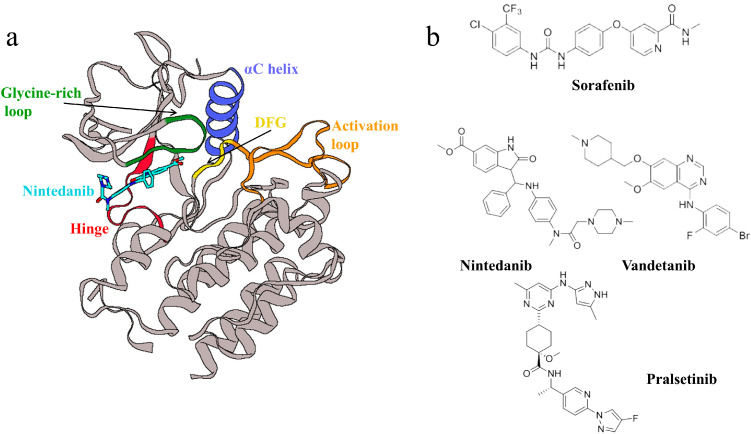
(**a**) The structure of RET, with important structural features highlighted in different colors. (**b**) Molecular structures mentioned in the main text.

RET (rearranged during transfection) is a receptor tyrosine kinase that participates in a multitude of complex biological processes, including intracellular and extracellular communication, cell growth, differentiation, and metabolism [1,2]. RET is also the receptor for the glial cell line-derived neurotrophic factor (GDNF) family. The binding of GDNF family ligands (GFLs) to GDNF family receptor-α (GFRα) mediates the dimerization of RET and its activation through the autophosphorylation of its kinase domain, which, in turn, activates multiple signaling pathways that play roles in the homeostasis and development of cells and tissues [3,4]. However, the abnormal activation of RET frequently results in oncogenesis, making it an important therapeutic target for various cancers. Initially discovered during a human proto-oncogene screen, the RET gene is commonly implicated in thyroid cancers, congenital megacolon, and non-small-cell lung cancers [5,6,7,8]. The RET kinase comprises a typical extracellular domain (ECD) at the N-terminus, a transmembrane domain (TM), an intracellular kinase domain, and a carboxy-terminal tail. The kinase domain is the primary binding site for inhibitors, and RET shares structural features common to other kinases: an N-terminal domain with five β-sheets and an α-helix (the αC-helix) and a C-terminal domain with six α-helices, connected by a hinge region that serves as the ATP binding site. The N-terminal domain also includes a glycine-rich loop (GRL), a DFG motif, and an activation loop, among others (Figure 1a) [9]. Several active tyrosine kinase inhibitors (TKIs) have been identified, primarily as multi-target inhibitors. Examples include Sorafenib, Vandetanib, and Nintedanib (Figure 1b) [9,10,11,12,13]. However, these drugs are multi-target inhibitors effective against RET with poor selectivity, and the development of effective, selective RET inhibitors is a top priority. Although the selective inhibitor Pralsetinib has been discovered, it has a certain degree of drug resistance [14]. Therefore, developing effective and selective novel RET inhibitors remains a significant challenge.

In the early stages of drug development, high-throughput screening is primarily used to identify active compounds that are either marketed or under development. The limited diversity of compound libraries makes it increasingly difficult to discover active compounds with novel scaffolds, especially receptor-specific compounds. Recently, artificial intelligence (AI) has been widely adopted in drug discovery research and development [15]. Deep learning models for molecule generation have gained widespread attention as a de novo design approach, providing the possibility of generating drugs for specific targets [16]. Generally speaking, the molecular generation models are usually based on an encoder–decoder approach, which explores the vast chemical space by encoding and decoding molecules in a latent space [17]. Several generative deep learning models proposed in recent years have achieved impressive results in generating new molecules. For example, Gómez-Bombarelli et al. applied a variational autoencoder (VEA) model based on the encoder–decoder architecture to identify molecules [18]. To improve molecular generation, Popova and Olivecrona et al. introduced memory augmentation, a recurrent neural network (RNN) structure, the architectures of which specialize in natural language processing (NLP) problems [19,20]. RNNs include bidirectional RNN (BRNN) [21], long short-term memory (LSTM) [22,23], Gate Recurrent Unit (GRU) [24], etc. As a special type of RNN, LSTM has not only feedforward neural networks but also feedback connections. Thus, compared to other traditional neural networks, LSTM performs very brightly in learning long-term dependencies, such as the simplified molecular-input line-entry system (SMILES) [25].

Despite the good performance demonstrated by generative deep learning (GDL) models in molecular generation, most GDL models are designed to generate the best possible molecules by predefined targets. The chemical structures of the generated molecules are more or less similar to those of known active compounds targeting the same objectives, not to mention the discovery of receptor-specific molecules. Therefore, fragment-based drug design (FBDD) is receiving attention as an effective molecular design method [26,27]. The core of FBDD is fragments, combined together with several strategies to generate easily synthesizable and more novel compounds. In other words, we generate molecules fragment by fragment instead of atom by atom. Because the chemical fragments are rational, the validity and diversity of the generated molecules can be improved as long as our connection approach is effective [28]. Thus, we built a chemical language model based on an LSTM encoder–decoder deep learning architecture and the FBDD method, which learns the sequence of the fragment assembly of active molecules and generates molecules with new scaffolds for the easier discovery of receptor-specific inhibitors. Our model also incorporates transfer learning to improve its specific generative capabilities. Finally, this model was applied to generate new RET-specific molecules that have chemical properties similar to known RET active molecules.

**Figure 2:**
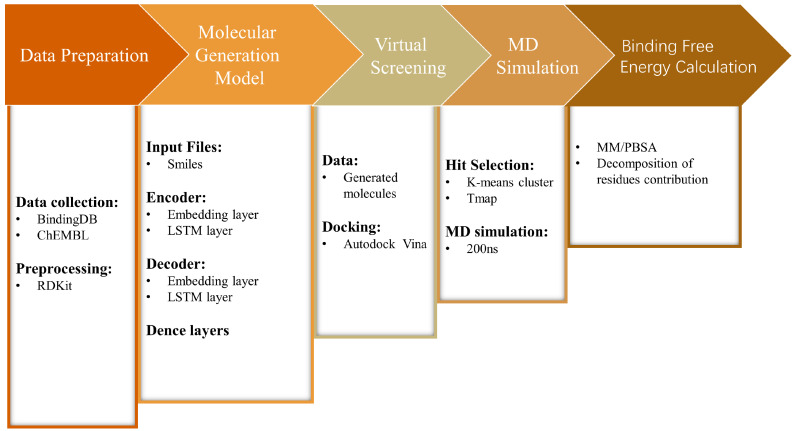
Flowchart of the methodology used in this study.

In this work, we constructed a molecular generation model based on an LSTM encoder–decoder deep learning architecture and the FBDD method to create a virtual molecular library for RET, and to perform virtual screening on this library (Figure 2). Molecules with relatively low binding energies were screened through clustering methods. Subsequently, the ADMET properties of these molecules were predicted and evaluated, and molecular dynamics simulations as well as binding free energy calculations were performed on the selected molecular systems and co-crystal complexes (RET–Pralsetinib). The results revealed that two of the generated molecules have similar interactions with the protein, compared with the co-crystal structure, and the binding energies of the generated molecules were even lower than those of the co-crystal structure. Therefore, we believe that our model can successfully identify potential selective RET inhibitors, providing new insights for the development of de novo drug design.

## 2. Results

### 2.1. Implementation and Evaluation of the Model

Traditional molecular generators usually add a generative model into the encoder–decoder structure to learn the distribution of latent vectors and generate new molecules. Because the training data are generally molecular sequences, this process leads to the generation of more or less similar molecules, making it difficult to identify receptor-specific molecules with novel scaffolds. Therefore, in this study, we used LSTM as the encoder and decoder, which is good at learning long-term dependency problems, and we combined it with the FBDD method, which treated molecular sequences as “sentences” and molecular fragments as “words”, as input to the LSTM encoder–decoder model. To obtain molecules with diverse scaffolds, we pre-trained the model with a large dataset of active molecules (~13 million) from the ChEMBL library to learn the splicing order of the active molecular fragments. To guide unbiased molecule generation focused on a specific chemical domain, after establishing the initial model, we used the corresponding active molecules (target data) to fine-tune it. Finally, we obtained a database containing 6369 molecules, with duplicates and molecules with unreasonable structures already removed.

**Figure 3:**
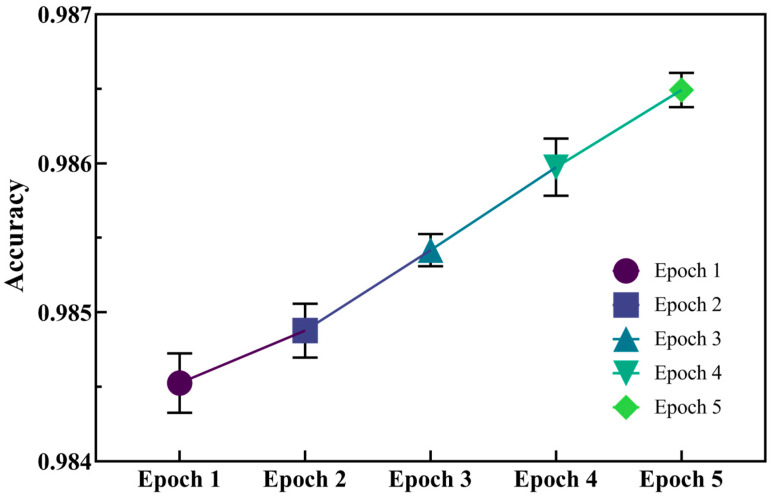
Accuracy of molecular fragment installation in five-round validation set.

To evaluate the capacity of molecular generation by the initial model, we used accuracy (Acc) as the metric (the closer to 1, the more accurate the model) to verify the model generation capabilities. As seen in Figure 3, five epochs were conducted, and the average accuracy of each epoch was above 0.984. As the epoch continued, the model accuracy was improved. This indicates that the initial generative model possesses exceptional generative capabilities, and that the molecules it produces are highly precise and reliable. 

### 2.2. Transfer Learning

**Figure 4:**
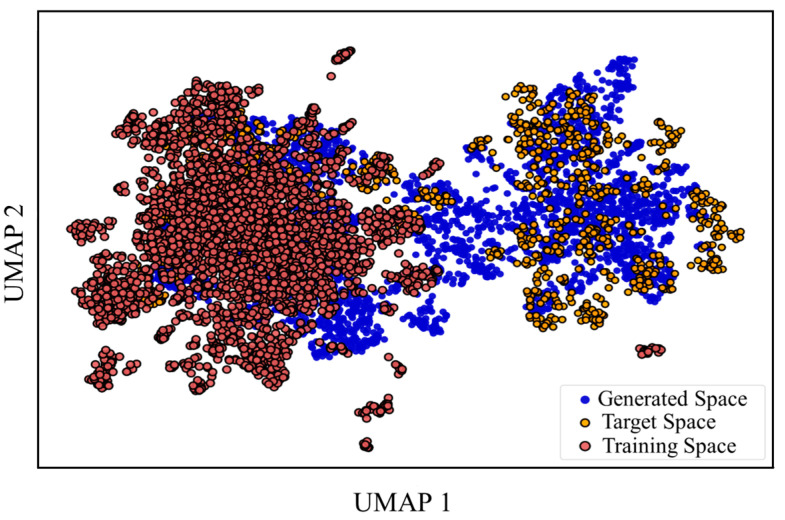
Chemical space distribution with transfer learning.

Because we wanted to build a well-performing model from limited known active compounds, we applied transfer learning. A large dataset containing about 13 million compounds (training data) was used to pre-train the initial model, and a dataset of RET active compounds (target data) was used to fine-tune it. To prove the effect of transfer learning, 10,000 random molecules from the training data, 3450 from the target data, and 6369 from the generated data were chosen to observe their chemical space similarity. The chemical space distribution of the training, target, and generated data was visualized using Uniform Manifold Approximation and Projection (UMAP) (Figure 4). The generated data molecules, post-fine-tuning, shifted from the training to the target, illustrating the effective navigational aspect of transfer learning from the training data to the target data. The result also demonstrates the ability to generalize the model, allowing for the migration of the initial model to other models with transfer learning.

**Figure 5:**
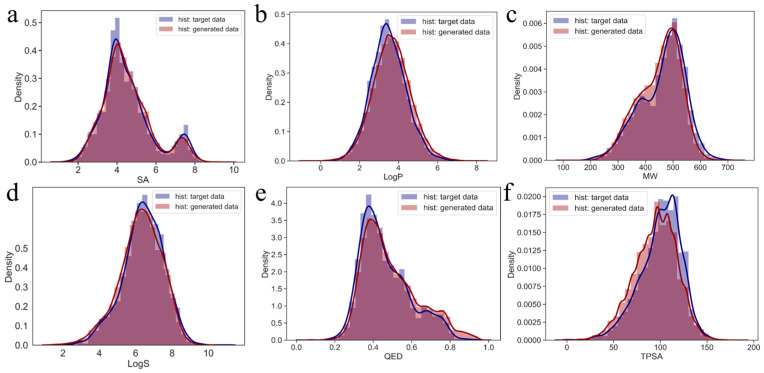
Kernel density estimation graphs for the generated and target data, represented in red and purple, respectively. (**a**–**f**) Kernel density estimation graphs for SA, LogP, MW, LogS, QED and TPSA of the generated and target data.

To further validate the relationship between the generated molecules in the virtual library and target molecules, their chemical property distributions (molecular weight (MW), octanol–water partition coefficient (LogP), quantitative estimation of drug-likeness (QED), water solubility (LogS), topological polar surface area (TPSA), and synthetic accessibility (SA)) were compared (Figure 5). The generated data and target data exhibit high similarity, indicating that the generated data share the same chemical properties as the target data, confirming that the molecules generated by the GDL model are chemically similar to the target molecules. Therefore, the molecules generated by this model can be considered candidate RET inhibitors.

### 2.3. Diversity of Generated Molecules

**Figure 6:**
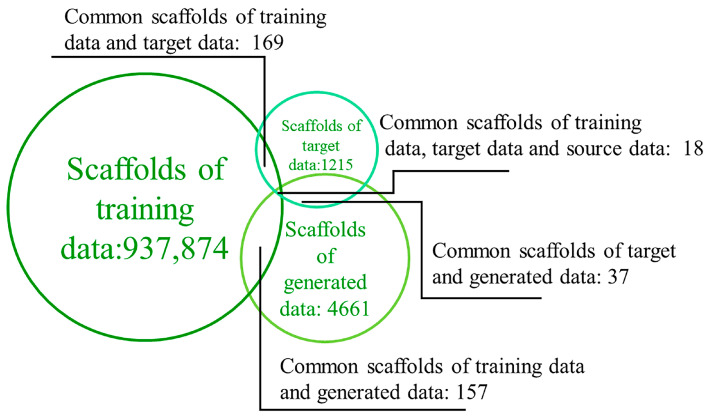
Numbers of scaffolds in the training, target, and generated data, and numbers of overlapping scaffolds.

The generation of novel scaffolds within the generated data was validated by calculating the number of molecular scaffolds. Although the number of Murcko scaffolds in the training data (937,874) was significantly higher than those in the generated (4661) and target (1215) data, it was found that very few scaffolds overlapped between the training and target/generated data (169 and 157, respectively) (Figure 6). Moreover, the generated data’s scaffolds not only included most of the target data’s scaffolds but also produced a large number of new scaffolds. In the generated data, 99.2% and 96.5% of the scaffolds were different from those in the target and training data, respectively. These results prove the model’s strong ability to generate additional scaffolds. All conclusions indicate that the GDL model can create a virtual library of molecules with chemical properties similar to the target and entirely novel scaffolds.

### 2.4. Virtual Screening

**Figure 7:**
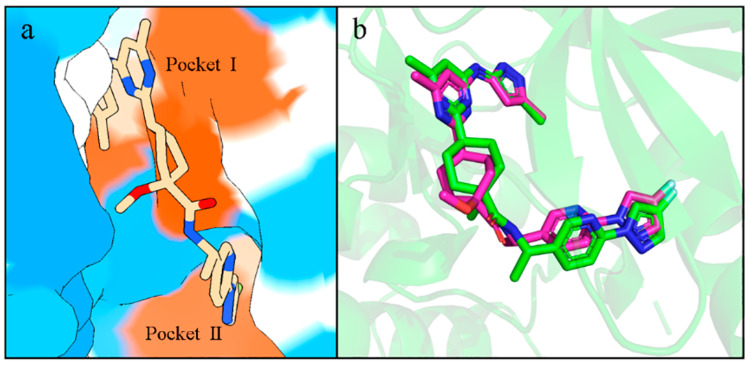
(**a**) Hydrophobic surface of RET with Pralsetinib, with redder colors indicating stronger hydrophobicity and bluer colors indicating stronger hydrophilicity. (**b**) Superimposition of the redocked conformation. The violet stick is the molecule in the crystal structure and the green stick is the re-dock molecule.

The generated molecules in the virtual library were batch-docked using Autodock Vina. Before the batch docking, the co-crystallized ligand containing Pralsetinib was redocked to the active site of RET and found to be highly congruent with the conformation and position of the co-crystal ligand, fitting into two hydrophobic pockets of RET (Figure 7a,b). This confirmed the reliability of our docking results. After establishing the reliability of the docking, approximately six thousand molecules from the virtual library were batch-docked. Considering that molecules with lower docking binding energies might have better activity, we selected molecules with the lowest affinities possible and excluded molecules with positive affinities.

**Figure 8:**
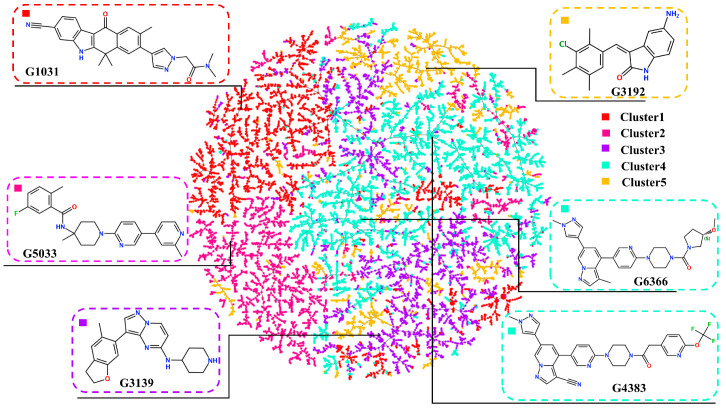
Clustering scatter plot for molecules with docking binding energies.

**Figure 9:**
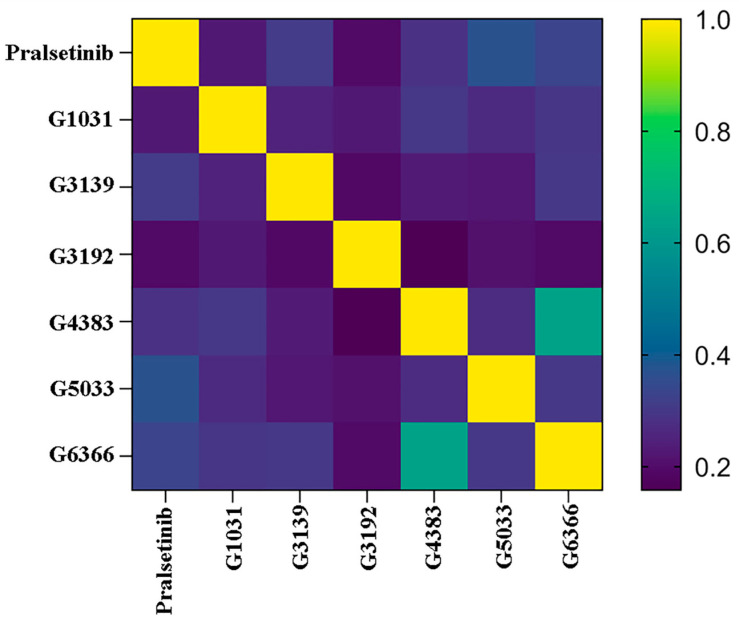
Heatmap of molecular similarity.

To exclude structurally similar analogs and increase the structural diversity, the remaining molecules were clustered using the K-means method after dimension reduction, forming five clusters (Figure 8). To select the most potential active molecules, one molecule with a relatively low docking binding energy was selected from each cluster for further studies. During the selection process, it was noted that the molecules in Cluster 4 generally had lower docking binding energies than those in the other clusters. Therefore, an additional molecule was selected from Cluster 4 to verify whether the molecules in Cluster 4 bind better to the RET kinase. To demonstrate the diversity of the selected molecular scaffolds, the similarity of seven molecules, including Pralsetinib, was assessed. As shown in Figure 9, apart from molecules G4383 and G6366, the similarity between the other molecules was around 0.2, and the similarity between G4383 and G6366 was around 0.6 because both molecules belonged to the same cluster. Moreover, the selected molecules are different from the scaffolds of all known RET-active compounds (Figure 6). Hence, we believe that the molecules selected from different clusters possess scaffold diversity.

### 2.5. ADMET Predictions

Using SwissADME and ADMETlab2.0, we predicted the ADMET properties of the six selected compounds, with the results presented in Table 1. All molecules fulfill Lipinski’s rule of five for drug-likeness, exhibit good synthetic feasibility, and have synthetic accessibility (SA) scores less than 6 (the smaller the number, the easier it is to synthesize), indicating ease of synthesis. The physicochemical properties of the selected molecules indicate lipophilicity (LogP) within the range of 0.7–6.0, signifying hydrophobicity conducive to entering the hydrophobic pockets of RET. The solubilities (LogS) of all selected molecules are below −6, indicating solubility (where insoluble is <−10, poorly soluble is between −10 and −6, and soluble is ≥−6). Regarding human intestinal absorption (HIA), all molecules are likely to be absorbed in the gut, while their skin permeabilities are low (the more negative the LogKp, the less skin permeation). For the toxicity parameter AMES, the highest probability of a molecule being positive is 21.2%, with the lowest at 6.4%, suggesting that these molecules are likely non-toxic according to AMES. Overall, the ADMET predictions indicate that our selected inhibitors possess favorable ADMET properties.

### 2.6. Molecular Dynamics Simulations

**Figure 10:**
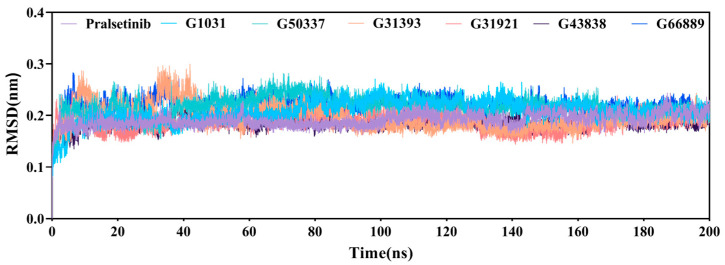
RMSDs of the seven complex systems.

Molecular dynamics (MD) simulations of 200 ns were conducted for the six generated molecules and the co-crystal structure (PDB ID: 6NEC), resulting in seven protein–ligand complex systems. The root-mean-squared deviation (RMSD) values of the protein–ligand complexes were calculated to observe the stability of the protein-ligand complexes. As shown in Figure 10, all seven complexes reached a stable state at around 40 ns, with fluctuations less than 0.3 nm. Aside from slight fluctuations in the G3139 system at around 60 ns, which then stabilized, all systems formed stable complexes until the end of the simulation. The RMSD results suggest that the six screened molecules can stably reside within the complex systems.

### 2.7. Binding Free Energy

The Molecular Mechanics/Poisson–Boltzmann (Generalized Born) Surface Area (MM/PBSA) method is an effective method for calculating the binding affinity between compounds and proteins, with lower free energy indicating more favorable stable binding. Considering that longer simulation times can produce more stable conformations, we used the last 10 ns out of a 200 ns trajectory, taking 500 frames for the binding free energy calculation. Upon computation, the binding free energy of the Pralsetinib co-crystal structure was found to be −30.580 kcal/mol and served as a benchmark. As shown in Table 2, all five generated molecules, except G3139, demonstrated lower binding free energies than the co-crystal structure during the simulation, with the lowest binding free energy being −40.015 kcal/mol (G6366). This suggests that all the generated molecules, except G3139, were tightly bound to the target protein in the simulation process. Further observation revealed that the van der Waals energy contributions were far more favorable than other contributions and played a crucial role in maintaining the complex stability, likely due to the formation of abundant hydrophobic interactions as the inhibitor molecules bound to the protein during the simulation. Based on the above analysis, we speculate that the high free energy of the G3139 complex system may be due to the fact that the key bond between the molecule G3139 and the receptor protein is not formed. Therefore, the result indicates that five molecules have good binding free affinities.

**Figure 11:**
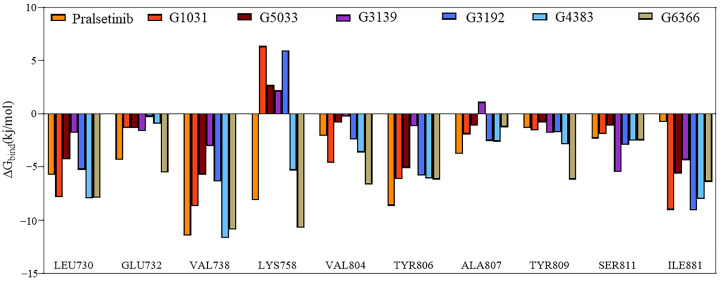
Energy contribution decomposition for the seven systems.

To verify our suspicions, residue binding free energy decomposition was performed. The decomposition results revealed that most residues contributed to the free energy in a trend consistent with the co-crystal structure, except for residues Lys758 and Ala807 (Figure 11). Residue Ala807 showed an unfavorable contribution in the G3139 complex system, which could be the reason for its higher binding energy in the MM/PBSA. The unfavorable contributions of residue Lys758 in the G3139, G3192, G1031, and G5033 complex systems suggested that these four systems did not form any interactions with residue Lys758 in RET. In contrast, residue Lys758 in the Pralsetinib, G4383, and G6366 complexes exhibited a strong, favorable contribution. Hence, the different energy contributions of Lys758 across the seven systems are probably due to different interactions between Lys758 and RET. Therefore, residues Ala807 and Lys758 make significant contributions to the binding free energy, which is the reason for the different free binding energies in the MM/PBSA.

### 2.8. Interactions of Generated Molecules with RET

**Figure 12:**
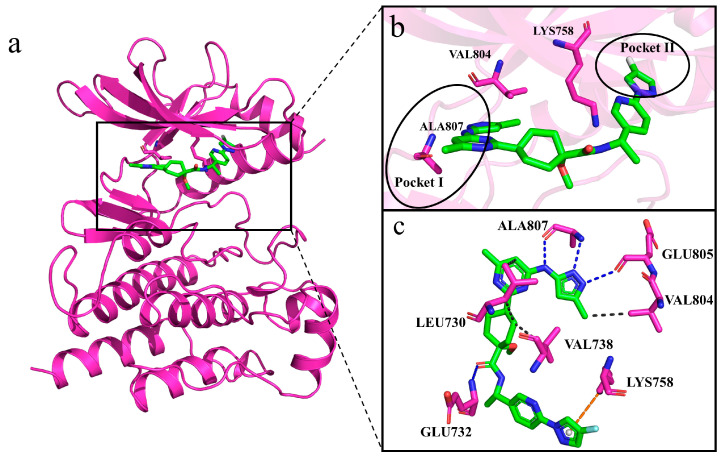
Interactions between Pralsetinib and RET. (**a**–**c**) Generated molecules are depicted using green sticks, RET protein is depicted as violet cartoon, and interacting residues are depicted as violet sticks. In panel (**c**), hydrogen bonds are illustrated with blue dashed lines, hydrophobic interactions with black dashed lines, and pi–cation interactions with orange dashed lines.

To further understand the binding mode of the molecules with the RET kinase, we first observed the interactions between Pralsetinib and RET (co-crystal structure) during the simulation, which served as a reference (Figure 12a–c) [29]. The contact surface between the receptor and protein primarily consists of Leu730, Glu732, Val738, Lys758, Val804, Glu805, and Ala807. RET forms hydrophobic interactions with Leu730 and Val738, hydrogen bonds with Glu732, Val804, Glu805, and Ala807, and a pi–cation interaction with Lys758. Pralsetinib mainly binds to Pocket I, the ATP binding site, including the hinge residues Val804, Glu805, and Ala807, to stabilize the compound in the pocket (Figure 12c). Within the complex systems, residue Ala807 is situated in the hinge region, and it forms hydrogen bonds with ATP and its competitive inhibitors to stabilize their presence in the pocket, making this residue extremely significant [30].

**Figure 13:**
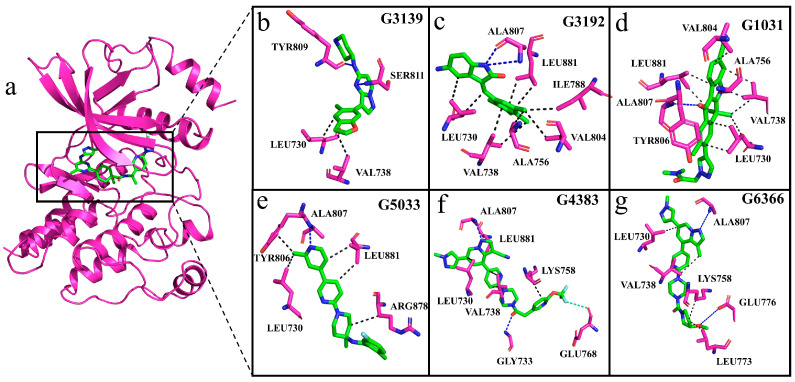
Interactions between generated molecules and RET. (**a**–**g**) The violet cartoon structure represents RET; violet sticks represent interacting residues; green sticks represent generated molecules; black dashed lines indicate hydrophobic interactions; blue dashed lines represent hydrogen bonds; and light-green dashed lines indicate halogen bonds.

We compared Pralsetinib with the generated molecular complex systems to determine the interaction of the generated molecules with RET. Observation of the interactions between the generated molecules and protein during the simulation reveals that, with the exception of molecule G3139, the other molecules are capable of forming hydrogen bonds with residue Ala807 (Figure 13a–g). To ascertain the permanence of these hydrogen bond interactions, hydrogen bond occupancy analysis was conducted throughout the entire simulation process. The data presented in Table 3 show that, apart from molecule G3139, the hydrogen bond occupancy rates with residue Ala807 exceed 50% for the generated molecules, indicating a stable hydrogen-bonding capacity with Ala807. In the MM/PBSA analysis, residue Ala807 showed an unfavorable contribution in the G3139 complex system, which aligns with our interaction analysis, mainly because G3139 was unable to form a hydrogen bond with residue Ala807 in RET, reaffirming the pivotal role of residue Ala807 in the stable binding of inhibitors. The inability of the generated molecule G3139 to form hydrogen bonds with residue Ala807 and other residues in the hinge region results in a reduced binding affinity. This finding is also consistent with our RMSD analysis, which showed fluctuations in the G3139 system at around 60 ns.

### 2.9. Selectivity between Generated Molecules with RET

Because we wanted to discover molecules that are selective for RET, we further compared the interactions of Pralsetinib with the generated molecules. For the Pralsetinib–RET complex, residue Val 804, as the gatekeeper (the first residue of the hinge region), along with Lys758, prevent the inhibitor from entering Pocket II. Molecular insertion into Pocket II can enhance the potency and selectivity for kinase inhibitors (Figure 12b). Although Pralsetinib binds extensively to the ATP pocket, its tail forms a Π–cation interaction with Lys758, avoiding passing through the gate between Val804 and Lys758 into Pocket II. Instead, Pralsetinib wraps around residue Lys758 to enter Pocket II. This insertion mode avoids spatial steric clashes with Val804 and Lys758, allowing the inhibitor Pralsetinib easier access to Pocket II. This novel binding mode grants Pralsetinib improved selectivity. The generated molecules are analyzed next as a reference to assess their selectivity and binding capacities.

**Figure 14:**
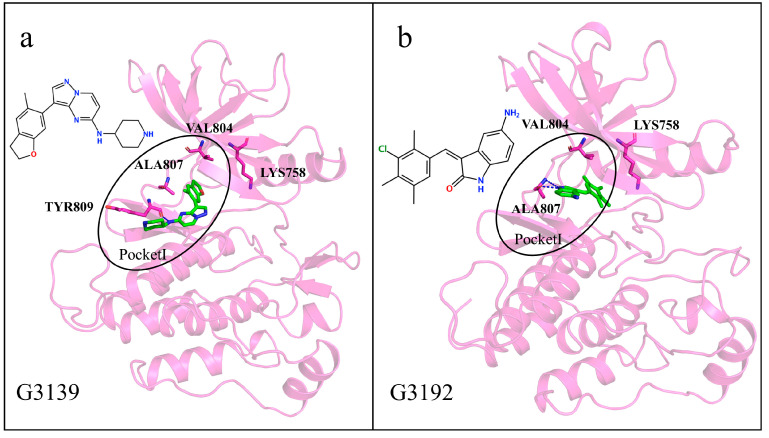
(**a**,**b**) Interaction diagrams of proteins with generated molecules. Generated molecules are represented by green sticks, RET protein is represented by violet cartoon, interacting residues are represented by violet sticks, and binding pockets are outlined in black circles.

To determine whether our generated molecules exhibit selectivity for RET, we discuss each of the six generated molecules. Firstly, we analyze the generated molecules G3139 and G3192, with relatively small molecular weights, which, due to their smaller molecular weights and volumes compared to other molecules, stably reside within the ATP binding pocket and are spatially similar to many multi-kinase inhibitors. They are distanced from the gatekeeper residues Val804 and Lys758 and are not likely to be inserted into Pocket II (Figure 14a,b). Although G3192 exhibited a lower binding energy than the co-crystal molecule, it also remained confined to Pocket I due to the hydrogen bonding of the residue Typ809. Thus, these two molecules are unlikely to exhibit the selective inhibition of the RET kinase. We considered that a generated molecule must have the potential to interact with the gatekeeper residues to be considered selective.

**Figure 15:**
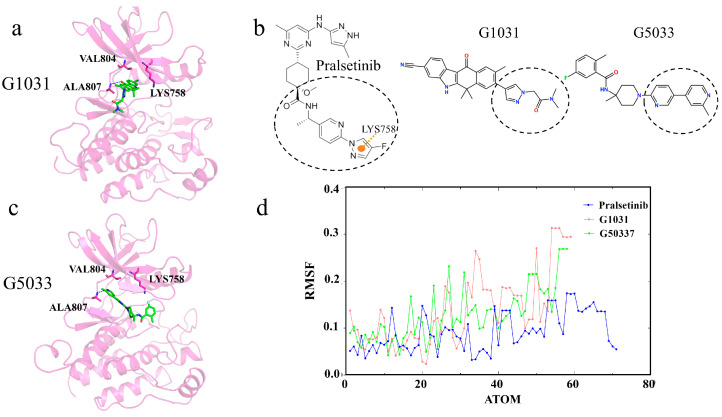
(**a**,**c**) Interaction diagrams of proteins with generated molecules. Generated molecules are represented by green sticks, RET protein is represented by violet cartoon, and interacting residues are represented by violet sticks. (**b**) Two-dimensional structure of the molecule. The pi–cation interaction is indicated with orange dashed lines. (**d**) Root-mean-squared fluctuations (RMSFs) of the generated molecules during the simulation.

Next, we analyze the generated molecules G1031 and G5033, which, compared to G3139 and G3192, are relatively larger and form hydrogen bonds with the hinge residue Ala807. They also have lower binding energies than the Pralsetinib complex and might stably bind in the ATP pocket (Figure 15a,c). However, neither molecule interacted with residues Val804 and Lys758; instead, they inserted themselves straight into Pocket I, consistent with the decomposition results of the residue free binding energy contributions. Observing Pralsetinib’s binding mode, only by adopting a “C”-shaped conformation and enveloping residue Lys758 can a molecule avoid the gatekeeper residues and have the opportunity to enter Pocket II (Figure 12b). Therefore, we considered that the key to maintaining a “C” shape is forming interactions with residue Lys758. Due to the formation of interactions with Lys758, Pralsetinib’s tail end is less flexible than the other two molecules, facilitating the maintenance of the “C” shape (Figure 15b,d). Hence, the generated molecules G1031 and G5033 also lack selectivity.

**Figure 16:**
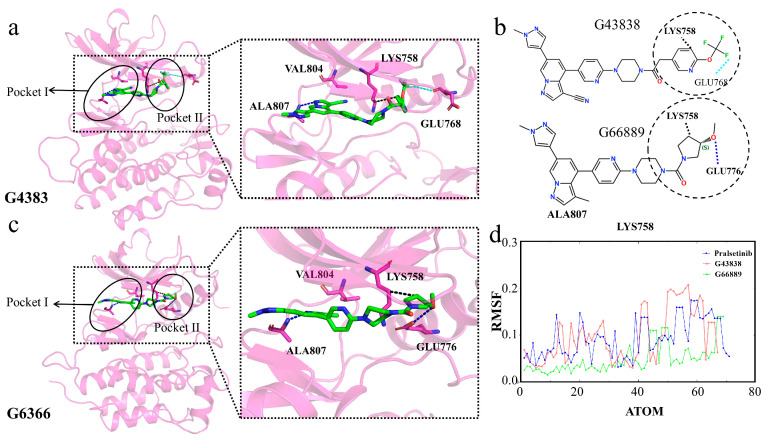
(**a**,**c**) Interaction diagrams of proteins with generated molecules. Generated molecules are represented by green sticks, RET protein is represented by violet cartoon, interacting residues are represented by violet sticks, binding Pocket I and Pocket II are outlined in black circles. (**b**) Two-dimensional structure of the molecules. (**d**) RMSFs of the generated molecules during the simulation.

Finally, we analyze the two molecules G4383 and G6366 from the same cluster. The results show that both molecules form hydrophobic interactions with residue Lys758 (Figure 16a–c). The formation of these interactions causes the molecules’ tails to bend similarly to Pralsetinib, allowing the tails to enter into Pocket II. Although G4383 and G6366 did not interact with the gatekeeper residue Val804, they still circumvented the gatekeeper residues Val804 and Lys758 of Pocket II by wrapping around Lys758 and entering the second active pocket. Throughout the simulation, the flexibility of the tails of G4383 and G6366 was similar to Pralsetinib, indicating that, during the simulation, the tails of the two molecules were fixed by residue Lys758, also adopting a “C” conformation to enter into Pocket II (Figure 16a,c,d). This conformation aligns with the Pralsetinib–RET complex. This interaction analysis is consistent with the MM/PBSA, and residue Lys758 is significant in the selectivity of the molecule. Therefore, we suggest that the generated molecules G4383 and G6366 exhibit selectivity.

## 3. Discussion

In this study, we employed a generative model to create a virtual library specific to RET for screening specific RET inhibitors. Subsequently, over six thousand molecules were virtually screened, with six molecules selected for further investigation through molecular docking, pharmacokinetic property prediction, and cluster analysis. Molecular dynamics (MD) simulations were conducted on the selected molecules and a co-crystal molecule containing Pralsetinib. The analysis of the simulation results revealed that the five compound systems were stable, serving as a basis for further research. The lowest energy conformation during the simulation was chosen for the analysis of molecular interactions with the protein. It was found that residues Ala807 and Lys758 play significant roles in the molecular binding process. Compound system G3139 did not form a hydrogen bond with Ala807, resulting in excessive system energy. Compound systems G3192, G1031, and G5033 did not interact with Lys758, leading to a lack of selectivity in the molecules. Only compound systems G4383 and G6366 interacted with both Ala807 and Lys758, maintaining a “C”-shaped conformation, allowing the molecule to bypass the gatekeeper residue and enter Pocket II, thereby increasing its selectivity. These results were also corroborated by residue contribution decomposition diagrams of the binding free energy. Based on these analyses, it is believed that the GDL model can produce selective candidate inhibitors that hold potential as new RET kinase inhibitors.

## 4. Materials and Methods

### 4.1. Preparation of the Dataset

A dataset of compounds from the ChEMBL database (https://www.ebi.ac.uk/chembl/, accessed on 6 February 2023) was downloaded as training data. A dataset of annotated RET activity compounds was retrieved from the BindingDB database (https://www.bindingdb.org/, accessed on 6 February 2023) as target data. Using the RDKit package v2020.09.1.0, these molecules were deduplicated, the stereochemistry was removed, the salts were stripped, and the SMILES strings were standardized, resulting in a training dataset of approximately 13 million molecules and a target dataset of 3450 active molecules.

### 4.2. Molecular Fragmentation

**Figure 17:**
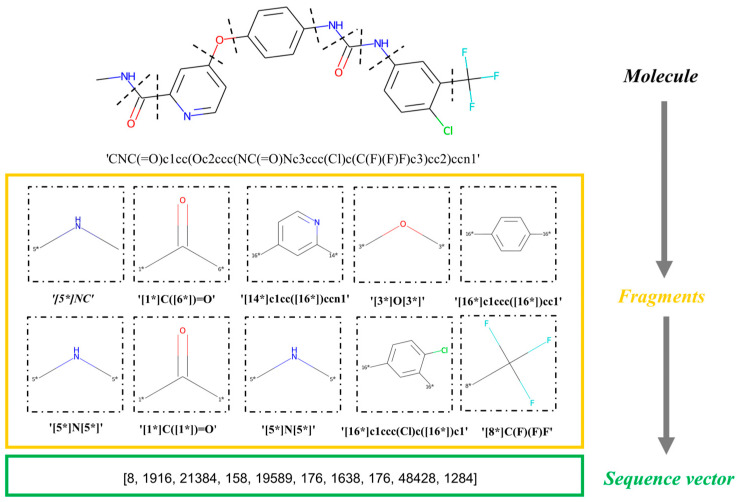
Flowchart of the molecule fragmentation process. “*” represents “Dummy” atom.

First, our model entails breaking the dataset into a sequence of fragments. Using the Breaking of Retrosynthetically Interesting Chemical Substructures (BRICS) algorithm in RDKit, a molecule is split into several fragments based on whether or not it can be synthesized [10], and each fragment is transformed into a standardized SMILES format, generating a set of fragment sequences. Briefly, the BRICS rules mean that molecular components with important functions and structures are retained. This algorithm scans the sequences of the SMILES ordered from left to right, extracting a fragment if a breakable bond is found. “Dummy” atoms are marked at the position of the cleavage sites. Our model uses molecular fragments as the basic units of input instead of strings [31]. The sequence of the fragments is viewed as a “sentence” and each fragment is viewed as a “word”. Each molecule in the dataset is converted into a fragment sequence representation, and after one-hot encoding, word-embedding processing, and batch processing, is input into our model. Note that the process of fragment extraction is in the left-to-right order according to the sequence of the SMILES. This process is fully reversible. In Figure 17, we display an example to illustrate the fragmentation and conversion into sequence vectors with our algorithm [32].

### 4.3. Construction of the Chemical Language Model

The chemical language model adopts an encoder–decoder approach, consisting of two LSTMs that act as the encoder and decoder. LSTM is a recurrent neural network technology that specializes in recognizing sequences, such as the SMILES. LSTM possesses a cell state, a hidden state, and the gates (a forget gate ($f_{t}$) is used to adjust the degree of retention of the cell state in the previous step; an input gate ($i_{t}$) is used to regulate the acceptance of inputs; and an output gate ($o_{t}$) is used to filter the output content). The cell state is called long-term memory, and the hidden state is called short-term memory, which are the origins of the name long short-term memory. Thus, LSTM performs well in dealing with sequences and temporal relationships between sequences. Here, we show the architecture in detail.

**Training:** The overall architecture follows the encoder–decoder scheme shown in Figure 4. This encoder–decoder is built using LSTM units, with two input layers, two embedding layers, two LSTM recurrent layers (512-dimensional), and one output layer.

**Figure 18:**
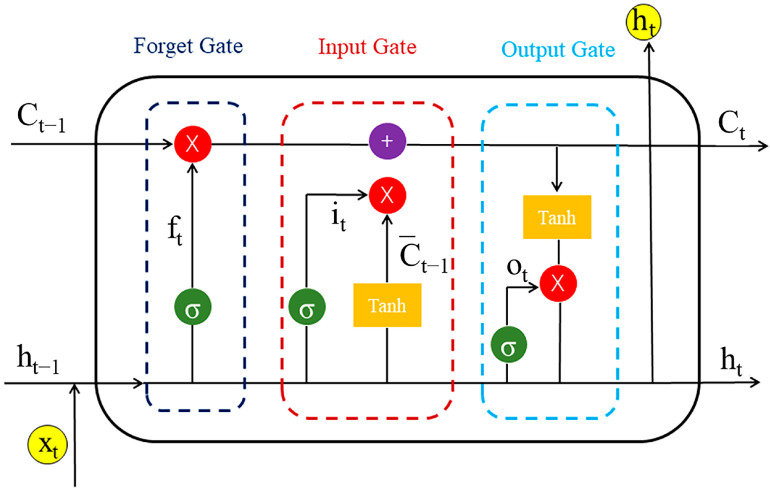
Flowchart of the LSTM algorithms.

Encoder: Input data (source data) are processed, and the embedding layer outputs a 512-dimensional initial vector as a conditional input for training in the LSTM. The encoder receives the source sequence of molecular fragments ($x_{i}$) and outputs the encoded state parameters (end [$h_{t\_e}$,$c_{t\_e}$]). The encoder is trained by LSTM algorithms as follows:

(1) $$f_{t}=\sigma (W_{f}\cdot \left[h_{t-1},x_{t}\right]+b_{f})$$

(2) $$i_{t}=\sigma (W_{i}\cdot \left[h_{t-1},x_{t}\right]+b_{i})$$

(3) $$o_{t}=\sigma (W_{o}\cdot \left[h_{t-1},x_{t}\right]+b_{o})$$

(4) $${\bar{C}}_{t}=\tanh\left(W_{c}\cdot \left[h_{t-1},x_{t}\right]+b_{c}\right)$$

(5) $$C_{t}=f_{t}C_{t-1}+i_{t}{\bar{C}}_{t}$$

(6) $$h_{t}=\tanh\left(C_{t}\right)$$

where $h_{t}$ is the hidden vector, $c_{t}$ is the memory state vector, and W is the weight matrix (Figure 18).

Decoder: The decoder receives the input fragment sequence and [$h_{t\_e}$,$c_{t\_e}$] passed on from the encoder, and it outputs the decoder output and [$h_{t}$,$c_{t}$]. Unlike the encoder, the decoder also calculates a new probability distribution for the output by stochastic sampling. To control the magnitude of randomness in the sampling process, we used temperature sampling as follows:

(7) ρi=1Qe−εikT=e−εikT∑j=1Me−εikT

where $\rho_{i}$ is the probability of state i; $\varepsilon_{i}$ is the energy of state i; k is the Boltzmann’s constant; T is the temperature of the system; and M is the number of all quantum states that the system can reach.

**Figure 19:**
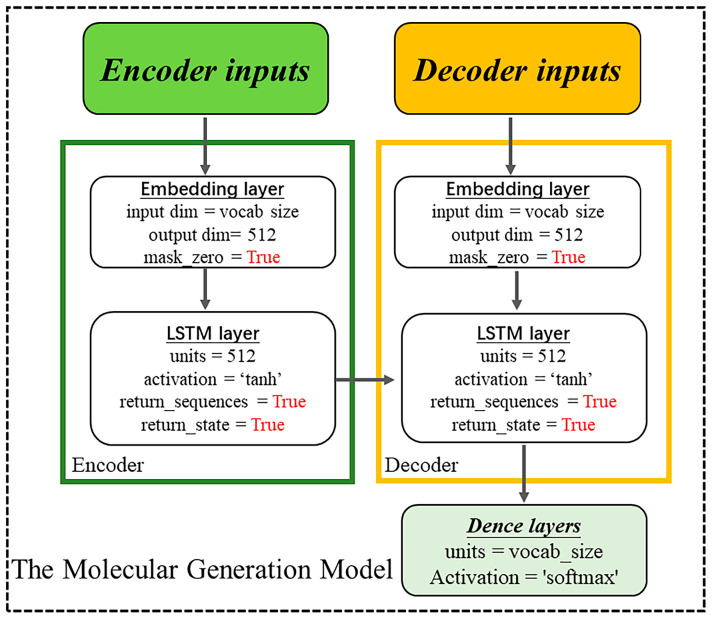
Model construction process flowchart.

**Generation:** In the generative process, we sample a latent vector as the first token of the model by stochastic sampling and input target sequences as fine-tuned data to construct a well-performing model. They are passed through the LSTM units and the output layer to produce the probability for the next fragment. The output layer is a dense, fully connected layer that receives the decoder output from the decoder and outputs a sequence vector processed by the softmax activation function. Finally, the resulting fragments are assembled into molecules according to the trained model sequence. The fragment sequences are standardized to a fixed length of 20, with padding of “0” for sequences shorter than this length. In the LSTM recurrent layer, the hidden layer count is 512, with the embedding layer containing 300,000 tokens and with an embedding dimension of 512. All the parameters and programs were implemented in Python v3.8, Tensorflow v1.1.4 [33], and Keras v2.2.5. Figure 19 displays the overall process of the model.

### 4.4. Model Evaluation

The preprocessing generated data were used to evaluate the generative capacity of the GDL model. To assess the generative capacity of the model, the dataset was split into a training set and validation set at a 0.75:0.25 ratio, with the accuracy (Acc) used as a model evaluation metric (8). True-positive (TP), false-positive (FP), false-negative (FN), and true-negative (TN) predictions can be obtained from the confusion matrix. The Acc is calculated using the following equation:

(8) $$Acc=\frac{TP+TN}{TP+FP+TN+FN}$$

The closer this parameter is to 1, the higher the accuracy of the model.

### 4.5. Molecular Property Calculation

To compare the similarity of various properties between the target data and generated data molecules, properties such as the molecular weight (MW), quantitative estimation of drug-likeness (QED), water solubility (LogS), octanol–water partition coefficient (LogP), topological polar surface area (TPSA), and synthetic accessibility (SA) were included. Kernel density estimation (KDE) plots and histograms were drawn using Seaborn to visualize the comparison results. All calculations were performed using RDkit. To further visualize the similarity of the chemical space distribution between the training data, target data, and generated data, the UMAP method was applied for dimensionality reduction and visualization. Due to the large volume of data, 10,000 molecules were extracted from the training for multiple rounds of dimensionality reduction and visualization, yielding consistent results.

### 4.6. Molecular Docking and Clustering

We conducted the docking of the generated 6369 molecules using Autodock Vina v1.2.2 software, referencing the crystal structure (PDB: 6NEC). Prior to docking, missing protein residues and atoms were completed using Jackal v1.0 software, and the protein was preprocessed by adding polar hydrogens. Consequently, Autodock tools were applied to prepare the ligand by keeping the number of rotatable bonds less than 6. The active-site grid was generated using the x, y, and z coordinates of the active site. The grid box center was positioned at the location of the small molecule within the crystal structure, with the box dimensions set to 18 Å × 18 Å × 18 Å with a grid spacing of 0.375 Å. The docking process was executed using the default parameters, and each molecule generated 10 poses. Following docking, molecules were clustered using the K-means method [34]. Based on the obtained contour coefficients, it was determined that the classification into five clusters is the best. Finally, the TMAP method was employed to visualize the five categories [35].

### 4.7. ADMET Prediction

The absorption, distribution, metabolism, excretion, and toxicity (ADMET) of drugs are critically important in contemporary drug design and screening. ADMET prediction is a fundamental criterion for assessing drug-likeness properties. We used two online prediction tools, SwissADME (http://www.swissadme.ch/, accessed on 10 June 2023) [34] and ADMETlab 2.0 (https://admetmesh.scbdd.com/, accessed on 10 June 2023) [36], to predict the ADMET characteristics of the generated molecules.

### 4.8. Molecular Dynamics Simulation

The Gromacs v2020 software package was utilized for molecular dynamics simulations. Before simulation, the seven molecules (six generated and one from the co-crystal structure) were first optimized using the Gaussian v09 software [37,38,39]. The processed small molecules and protein were used to build the protein–ligand complex system. The system was solvated using the Amber force field and TIP3P water model, and Na+ and Cl− were added as counterions to neutralize the system’s charge [40]. Energy minimization was then performed using the steepest descent method to obtain the lowest energy conformation. Equilibration was carried out at 310 K using the V-rescale method for 500 ps under NVT conditions. Under 1 atmosphere of pressure, 500 ps of NPT equilibration was conducted using the Parrinello–Rahman algorithm. Finally, a 200 ns dynamics simulation was performed. The LINCS algorithm was applied to constrain bonds during the simulation, and the Particle Mesh Ewald (PME) method was used for long-range electrostatics [41].

### 4.9. Binding Free Energy Calculation

Following the simulation, the gmx_MMPBSA tool (https://github.com/Valdes-Tresanco-MS/gmx_MMPBSA, accessed on 5 January 2023) was utilized to perform Molecular Mechanics/Poisson–Boltzmann Surface Area (MM/PBSA) binding free energy calculations [42]. The binding free energy (G_bind_) consists of three energy components: the vacuum potential energy (E_MM_), polar solvation energy (G_GB_), and nonpolar solvation energy (G_SA_). The E_MM_ includes bonded (bonds, angles, and dihedrals) and nonbonded interactions (electrostatic interactions ($E_{ele}$) and van der Waals interactions ($E_{vdw}$)), which are calculated using molecular mechanics parameters. The G_GB_ and G_SA_ are calculated using the Poisson–Boltzmann equation and the solvent accessible surface area (SASA), respectively [43,44]. The equation for the compound system in solution is as follows:

(9) $$G_{bind}=∆E_{MM}+∆G_{GB}+∆G_{SA}+-T∆S\;=∆E_{vdw}+∆E_{ele}+∆G_{GB}+∆G_{SA}-T∆S$$

TΔS represents the entropic contribution at temperature (T).

## Figures and Tables

**Table 1:** Drug-likeness predictions.

Generated Molecules	Physicochemical Properties		Pharmacokinetics		Toxicity	Drug-Likeness	SA
	LogP	LogS	HIA	LogKp	AMES	Lipinski Rule	
G3139	3.790	−3.75	0.996	−6.47 cm/s	0.937	Accepted	2.577
G3192	4.368	−4.41	0.997	−5.59 cm/s	0.946	Accepted	2.402
G1031	4.246	−5.952	0.976	−6.19 cm/s	0.788	Accepted	3.001
G5033	4.930	−4.208	0.997	−5.70 cm/s	0.837	Accepted	2.577
G4383	5.002	−5.17	0.996	−7.73 cm/s	0.931	Accepted	3.214
G6366	3.927	−4.299	0.996	−8.14 cm/s	0.956	Accepted	3.449

**Table 2:** Binding free energies (kcal/mol) of the seven complex systems.

System	ΔE_ele_	ΔE_vdw_	ΔG_PB_	ΔG_NP_	−TΔS	ΔG_bind_
Pralsetinib	−10.124	−63.360	47.151	−10.125	2.860	−30.580
G1031	−5.648	−55.550	27.913	−6.125	3.443	−35.568
G5033	−8.529	−44.978	25.027	−7.696	5.160	−31.016
G3139	−6.012	−39.480	21.128	−4.610	2.439	−26.535
G3192	−5.908	−45.231	22.457	−4.731	2.849	−30.565
G4383	−5.553	−65.031	32.635	−7.667	6.177	−39.441
G6366	−9.329	−68.719	14.055	−7.354	4.333	−40.015

**Table 3:** Occupancy rates and average numbers of hydrogen bonds during simulation.

Molecules	Residues	Occupancies
Pralsetinib	Ala807	94.2%
G1031	Ala 807	53.81%
	Asp892	22.23%
G5033	Ala807	62.8%
	Glu733	12.4%
G3139	Tyr809	50.3%
G3192	Ala807	80.8%
G4383	Ala807	85.1%
	Glu733	40.5%
G6366	Ala807	89.3%
	Glu766	50.6%

## Data Availability

The required data and codes of our model are available on GitHub (https://github.com/LuLu1991/LSTM-autoencoder, accessed on 3 January 2024).
